# Experience with pediatric sarcoidosis at a centre in Mumbai, India

**DOI:** 10.1186/1546-0096-9-S1-P37

**Published:** 2011-09-14

**Authors:** RP Khubchandani, RP Hasija, I Touitou, C Khemani

**Affiliations:** 1 Pediatric Rheumatology Clinic, Jaslok Hospital, Mumbai, India; 2 University Hospital Center of Montpellier, Montpellier Cedex 5, France

## Background

Pediatric Sarcoidosis is a rare multisystem granulomatous disorder and series from the Asian subcontinent are few.

## Aim

We describe our experience to date with an inceptional cohort.

## Methods

Retrospective chart review of the demographic, clinical, diagnostic and genetic characteristics were studied.

## Results

Over seven years, 12 of 1214 new cases seen in the Pediatric Rheumatology Clinic (1%) (M: F = 1:1), were diagnosed as Sarcoidosis. 11/12(91.7%) had an onset ≤4 years of age, 8/12(67%) maintained a cumulative follow-up of 33.7 years ( range 1-9 years). 7/12(58.3%) had received anti-tuberculous therapy prior to referral.

**Table:** 

Cohort features	Characteristics at Onset/ 1^st^ visit	Follow-up ( ≥ 1 year)
Number(N) Median age (years) [IQR]	12 Onset:1.25[0.9-1.7]Diagnosis:7.8[4.9-9.9]	8

Fever; median duration	10/12(83.3%);18 months	Resolution

Arthritis	7/12(58.3%)	Resolution

Skin manifestations	7/12(58.3%)	Resolved 6/8(75%)

Ocular abnormalities	Onset: 6/12(50%), 1st visit: 5/6(83.3%)	2/5(40%)

Triad-Arthritis,Rash,Uveitis	Onset: 5/12(41.7%), 1st visit: 3/5(60%)	None

Systemic features	Sicca(1),Adenopathy(4),GIT(4), Pulmonary(5), Organomegaly(9)	Aortoarteritis(1/8) Interstitial lung(1/8)

Growth retardation	12/12(100%)	5/8(62.5%)

Median Steroid dose(mg/kg/day) [IQR] Median Methotrexate dose(mg/m2BSA) [IQR] Others:	0.6[0.2-0.8] 10.1[0-10.4]	0.3[0.1-0.3] 10.9[9.7-15.3] Azathioprine(vasculitic rash)(1),Mycophenolate(uveitis)(1)

Treatment side-effects	-	Hepatotoxicity(1/8) Osteoporosis(2/8) Cataracts(3/8)

Diagnosis was by clinical presentation 'plus': ACE (4/12), biopsy (1/12), biopsy and ACE (3/12), biopsy and mutation (1/12), mutation (2/12). 3/9(33.3%) are positive for CARD15 mutation (Blau Syndrome). 2 have sporadic mutations at R334W while 1 with a mutation at G464W, developed cardiomyopathy and aortoarteritis and has a symptomatic parent with the identical mutation. None of the 8 patients following up are off therapy. 5/8(62.5%) achieved clinical improvement in a median duration of 6.9 months[5.6-9.6 IQR].

## Conclusions

In our setting, Pediatric Sarcoidosis had a significant time lag to diagnosis, being often initially diagnosed as tuberculosis owing to similar clinical picture and histology. Morbidity is considerable, with arthritis, fever and rash responding to therapy while eye changes and organ damage are relatively refractory. All children show significant growth retardation at diagnosis and follow up inspite of control of constitutional features. Amongst the 3 Blau Syndrome patients, one had an atypical presentation and an autosomal dominant inheritance.

